# Elucidating the binding mechanism of *Caralluma tuberculata* metabolites with type 2 diabetes targets through molecular docking and dynamics simulations

**DOI:** 10.1371/journal.pone.0343905

**Published:** 2026-04-03

**Authors:** Amir Ali, Zia-ur-Rehman Mashwani, Ashfaq Ahmad, Juan Pedro Luna-Arias, Gabriela Medina-Pérez, Ajaz Ahmad

**Affiliations:** 1 Department of Botany, PMAS Arid Agriculture University Rawalpindi, Pakistan; 2 Department of Cell Biology, Nanoscience and Nanotechnology Ph.D. Program, Center for Research and Advanced Studies of the National Polytechnic Institute, Mexico City, Mexico; 3 Institute of Agricultural Sciences, Autonomous University of the State of Hidalgo, Hidalgo, Mexico; 4 Department of Bioinformatics, Faculty of Natural and Computational Sciences, Hazara University, Mansehra, Pakistan; 5 Department of Clinical Pharmacy, College of Pharmacy, King Saud University, Riyadh, Saudi Arabia; Kwara State University, NIGERIA

## Abstract

**Introduction:**

*Caralluma tuberculata*, a medicinal plant from the Apocynaceae family, has been traditionally used to manage diabetes due to its rich secondary metabolite content.

**Methodology:**

This study employed LC/ESI-MS/MS analysis to identify bioactive compounds in *C. tuberculata*, followed by in silico screening for their inhibitory effects on key carbohydrate-digesting enzymes—alpha-amylase, sucrase, and alpha-glucosidase associated with type 2 diabetes. A total of 57 compounds were evaluated through molecular docking, toxicity prediction, drug-likeness analysis, and molecular dynamics (MD) simulations.

**Results:**

Among these, luteolin exhibited the highest binding affinities with amylase (−9.725 kcal/mol), sucrase (−8.19 kcal/mol), and alpha-glucosidase (−7.842 kcal/mol), while also demonstrating no predicted toxicity. MD simulations over 60 ns revealed stable root mean square deviation (RMSD) profiles for all protein-ligand complexes, confirming system stability. Free binding energy calculations (MM-PBSA and MM-GBSA) further suggested that luteolin had stronger, more stable interactions with amylase and sucrase compared to glucosidase.

**Conclusion:**

This study provides a comprehensive computational evaluation of luteolin derived from *Caralluma tuberculata*, offering detailed insights into its enzyme-specific interactions with key carbohydrate hydrolyzing enzymes relevant to type 2 diabetes. Although the results are promising, experimental and clinical validation is essential to confirm luteolin’s therapeutic efficacy for managing type 2 diabetes.

## Introduction

Type 2 diabetes mellitus (DM) is a chronic and increasingly prevalent condition worldwide, predisposing individuals to complications and heightened susceptibility to common infections [1]. This increased susceptibility can lead to complex conditions, including kidney damage, cognitive impairment, and cancer, ultimately contributing to greater morbidity and mortality rates [2]. Type 2 DM is marked by hyperglycemia and insulin resistance, with elevated blood glucose levels resulting from inadequate insulin production by pancreatic cells. One approach to managing hyperglycemia in type 2 DM involves inhibiting dietary carbohydrate metabolism [3]. Typically, dietary carbohydrates are broken down into monosaccharides by alpha-amylase in the digestive system and further converted to glucose by alpha-glucosidase for absorption into the bloodstream. Therefore, inhibiting these enzymes can reduce carbohydrate metabolism and lower blood glucose levels [4]. Type 2 DM treatment strategies often depend on patient characteristics, the severity of hyperglycemia, and available therapeutic options. Common oral medications include metformin, sulfonylureas, and thiazolidinediones, but long-term use of these drugs can lead to adverse effects such as liver complications, hypoglycemia, and gastrointestinal issues. As a result, there is a need for new antidiabetic agents with improved efficacy and lower toxicity. Herbal medicinal plants present a promising source for such drug development, as they typically offer lower toxicity, higher specificity, and greater availability [5]. The therapeutic potential of medicinal plants has spurred researchers to explore novel agents with minimal side effects [6]. Plants produce a variety of secondary metabolites, including terpenes, sterols, glucosides, flavonoids, tannins, and others, which not only support their growth but also serve as sources for potential new drugs [7].

Current strategies for developing new antidiabetic agents rely on traditional and ethnopharmacological knowledge of medicinal plants known to contain beneficial secondary metabolites [8]. Among such medicinal plants, the genus Caralluma, particularly *Caralluma tuberculata*, has a long history of use in traditional medicine for managing DM. Research suggests that the Caralluma genus is rich in bioactive compounds with antidiabetic potential [9]. *Caralluma tuberculata*, a species within this genus, is valued for its secondary metabolites, including terpenes, sterols, flavonoids, and glucosides, which exhibit significant antidiabetic properties. These bioactives may help reduce blood glucose levels by inhibiting enzymes like alpha-amylase, sucrase, and alpha-glucosidase, enhancing insulin secretion, and increasing glucose uptake [10]. The antidiabetic effects of *Caralluma tuberculata* are thought to arise from mechanisms such as stimulating pancreatic insulin secretion, enhancing insulin receptor activity, improving glucose tolerance, and reducing insulin resistance [11]. These findings imply that *Caralluma tuberculata* could help protect tissues from oxidative stress related to diabetes, though further metabolomic studies are necessary to fully understand its antidiabetic potential.

Liquid chromatography-mass spectrometry (LC/MS) is an advanced analytical technique widely used for identifying and quantifying bioactive compounds to meet pharmaceutical research needs [12]. Electrospray ionization (ESI), coupled with LC/MS, is used to ionize analyte molecules, allowing analysis based on their mass-to-charge ratio (m/z) [13]. This study uses LC ESI MS/MS to profile the phytochemicals in *Caralluma tuberculata*, generating mass spectra data valuable to researchers and industry stakeholders interested in developing therapeutic agents. To identify effective inhibitors from *Caralluma tuberculata*, 57 phytochemicals were isolated through LC/ESI-MS/MS and screened virtually against alpha-amylase, sucrase, and alpha-glucosidase enzymes using in silico approaches. Among these compounds, Luteolin demonstrated the highest binding affinity with amylase, sucrase, and alpha-glucosidase and exhibited zero toxicity, making it a promising candidate for drug development. Molecular dynamics simulations conducted over 60 ns confirmed stable RMSD patterns for all proteins, indicating a stable system. MM-PBSA and MM-GBSA calculations further validated that amylase and sucrase had stronger free binding energies than alpha-glucosidase. Overall, Luteolin formed more stable complexes with amylase and sucrase, while interactions with glucosidase were less stable. The significance of this work lies in providing a comprehensive enzyme specific computational analysis of luteolin in the context of *Caralluma tuberculata*, offering mechanistic insights that may support future experimental validation and drug development. Further, in vivo studies are necessary to confirm its efficacy and safety as a therapeutic agent for type 2 diabetes.

## Materials and methods

### Source of plant

*Caralluma tuberculata* was successfully cultivated in a greenhouse Agricultural Research Institute (ARI) Tarnab Peshawar, Peshawar 25000, Pakistan) under conditions simulating its native warm, semi-arid habitat. Day time temperatures were maintained at 25–28 **°**C with moderate humidity (60–70% during initial acclimatization) using a digital thermometer hygrometer (Model: HTC-1). Plants were grown in a well-draining sandy-loam or cactus soil mix (2 parts sand, 1 part loam, 1 part compost) and watered sparingly to prevent root rot, allowing the soil to dry slightly between irrigations. A commercial low nitrogen, potassium rich fertilizer (NPK 5–10–10 (Fauji Fertilizer Company Limited (FFC)) was applied every 2–3 weeks during the active growing season. Greenhouse cultivation provided protection from pests, grazing animals, and excessive rainfall, creating a controlled environment suitable for conservation and research. All procedures were conducted in accordance with relevant institutional, national, and international regulations, including permits for plant collection and cultivation, and in compliance with the Convention on Biological Diversity and applicable botanical research guidelines. The plant *Caralluma tuberculata* was identified by Botanist Dr. Zia Ur Rehman Mashwani. This plant material was used in my PhD Thesis project Notification Number (PMAS-AAUR/CE/12 and featured in my previous publication [14] The collection was conducted without harming the natural population of the species by follwoing the institutional ethical guidelines. specimen was deposited in a publicly accessible herbarium Quid Azam univeristy, Islamabad, Pakistan with Voucher Number RAW 103626. Only 2 g of fresh shoots (fresh weight) were harvested for LC–MS analysis to minimize environmental impact and support conservation for further propagation.

### LC/ESI-MS/MS spectrometry analysis

LCMS analysis of the antidiabetic compounds present in the 50% methanolic extract of *Caralluma tuberculata* was performed using a Thermo Electron Corporation LC–ESI–MS/MS system (Thermo Fisher Scientific, USA) equipped with an electrospray ionization (ESI) source operated in positive ion mode with a capillary spray source. A direct injection method was employed for compound detection. The spray voltage was set to 3.5 kV, with a sheath gas flow rate of 35 arbitrary units and an auxiliary gas flow rate of 10 arbitrary units. The capillary (ion transfer tube) temperature was maintained at 280 °C, and the sample flow rate was fixed at 8 µL/min. Mass spectra were acquired over an m/z range of 50–1,000. For tandem MS (MS/MS) analysis, collision induced dissociation (CID) energies were optimized and varied between 10 and 45 eV, depending on the physicochemical characteristics of the parent ions, to generate informative fragment ion spectra. All analytes were analyzed using identical source and instrumental parameters to ensure consistency and comparability. Data acquisition, processing, and spectral interpretation were carried out using Thermo Xcalibur software (version 4.2). Structural elucidation of detected metabolites was performed through a combination of manual spectral analysis, database comparison, and chemical structure visualization using ChemDraw Ultra 8.0, following previously reported protocols [15].

### Molecular docking analysis

To identify optimal binders from the sample extract, X-ray crystal structures of human α-amylase (PDB ID: 5EMY; resolution 1.23 Å), sucrase (PDB ID: 3LPP; resolution 2.15 Å), and α-glucosidase (PDB ID: 7KBJ; resolution 2.21 Å) were retrieved from the RCSB Protein Data Bank (https://www.rcsb.org/). For molecular docking, Chain A was used for α-amylase (5EMY), sucrase (3LPP), and α-glucosidase (7KBJ), as it contains the complete catalytic domain in each structure. The active sites were defined based on the coordinates of the co-crystallized ligands and experimentally reported catalytic residues. Docking validation was ensured by selecting receptor structures that retained their native co-crystallized ligands, thereby avoiding blind docking and enabling accurate definition of experimentally verified active sites. The native ligands glycosyl epi cyclophellitol (α-amylase), kotalanol (sucrase), and triazon (α-glucosidase) were preserved and used for grid box generation. Docking grids were centered on the catalytic sites of each enzyme using the native ligand coordinates, and all ligand poses were visually inspected to confirm interactions with key catalytic residues. A library of 57 phytochemical compounds was prepared by obtaining canonical SMILES from the PubChem database (https://pubchem.ncbi.nlm.nih.gov/) and converting them into three-dimensional structures using the RDKit package (https://www.rdkit.org/). Receptor preparation involved removal of crystalline salts, water molecules, and non-essential heteroatoms, followed by the addition of polar hydrogens and Gasteiger charges. Ligand structures were energy-minimized using the conjugate gradient method with the Universal Force Field (UFF) over 50,000 steps and a convergence threshold of 1 × 10 ⁻ ⁶. [16]. A convergence criterion of 0.000001 was applied, allowing the program to perform iterative cycles. The LaBOX method was employed to define the docking grid, centered on the coordinates of the co-crystallized ligands to ensure active-site-specific docking. Molecular docking was performed using AutoDock Vina (https://vina.scripps.edu/) with an exhaustiveness value of 16 [17]. Docking poses were evaluated based on binding affinity, pose consistency, and RMSD stability, and the ten highest-ranked compounds for each receptor were selected. Post-docking interaction analyses were carried out using PLIP to examine hydrogen bonding, hydrophobic contacts, salt bridges, π–π stacking, π–cation interactions, halogen bonds, and metal coordination [18].

### Property analyses of top binders

Our aim was to select a compound that not only demonstrated the best binding score and RMSD but also exhibited zero toxicity risk and favorable drug-like properties. We therefore, evaluated toxicity risk, cLogP (solubility), molecular weight (MW), drug score (DS), drug-likeness (DL), and TPSA. Toxicity risks were predicted in silico as preliminary indicators rather than confirmed toxicological outcomes. These assessments were conducted using libraries and open-source tools available at http://www.openmolecules.org. Among the top ten binders for each receptor, Luteolin (PubChem CID 5280445) emerged as the sole compound meeting criteria for low toxicity, acceptable cLogP, MW, TPSA, and drug-likeness, making it the most suitable candidate across all three receptors. Detailed evaluations are presented in Table 1.

**Table 1:** Library of 57 compounds identified in LC-MS analysis. All the compounds are linked to the PubChem identifier and canonical SMILES.

Compound Name	PubChem CID	Canonical-Smiles
Rutin	5280805	C[C@H]1[C@@H]([C@H]([C@H]([C@@H](O1)OC[C@@H]2[C@H]([C@@H]([C@H]([C@@H](O2)OC3 = C(OC4 = CC(=CC(=C4C3=O)O)O)C5 = CC(=C(C = C5)O)O)O)O)O)O)O)O
Kaempferol-7-O-alpha-L-rhamnopyranoside	25079965	C[C@H]1[C@@H]([C@H]([C@H]([C@@H](O1)OC2 = CC(=C3C(=C2)OC(=C(C3 = O)O)C4 = CC = C(C = C4)O)O)O)O)O
Catechin (phenol)	289	C1 = CC = C(C(=C1)O)O
Ferulic acid	445858	COC1 = C(C = CC(=C1)/C = C/C(=O)O)O
Coumarin	323	C1 = CC = C2C(=C1)C = CC(=O)O2
Pregnane	6857422	CC[C@H]1CC[C@@H]2[C@@]1(CC[C@H]3[C@H]2CCC4[C@@]3(CCCC4)C)C
Flavone	10680	C1 = CC = C(C = C1)C2 = CC(=O)C3 = CC = CC = C3O2
Quercetin	5280343	C1 = CC(=C(C = C1C2=C(C(=O)C3 = C(C = C(C = C3O2)O)O)O)O)O
Kaempferol 3-O-beta-D-glucoside	5282102	C1 = CC(=CC = C1C2=C(C(=O)C3 = C(C = C(C = C3O2)O)O)O[C@H]4[C@@H]([C@H]([C@@H]([C@H](O4)CO)O)O)O)O
Gallic acid	370	C1 = C(C = C(C(=C1O)O)O)C(=O)O
Caffeic acid	689043	C1 = CC(=C(C = C1/C = C/C(=O)O)O)O
Reducing sugars	11873	C1 = C(C = C(C(=C1C(=O)O)O)[N+](=O)[O-])[N+](=O)[O-]
3-O-Beta-D-Glucopyranosyl-Beta-D-Glucopyranose	5287770	C([C@@H]1[C@H]([C@@H]([C@H]([C@@H](O1)O)O)O[C@H]2[C@@H]([C@H]([C@@H]([C@H](O2)CO)O)O)O)O)O
kaempferol 3-O-beta-D-glucopyranosyl-7-O-alpha-L-rhamnopyranoside	21606527	C[C@H]1[C@@H]([C@H]([C@H]([C@@H](O1)OC2 = CC(=C3C(=C2)OC(=C(C3 = O)O[C@H]4[C@@H]([C@H]([C@@H]([C@H](O4)CO)O)O)O)C5 = CC = C(C = C5)O)O)O)O)O
luteolin 4’-O-beta-D-glucoside	5320844	C1 = CC(=C(C = C1C2=C(C(=O)C3 = C(C = C(C = C3O2)O)O)O)O)O[C@H]4[C@@H]([C@H]([C@@H]([C@H](O4)CO)O)O)O
Emodin	3220	CC1 = CC2 = C(C(=C1)O)C(=O)C3 = C(C2 = O)C = C(C = C3O)O
5.alpha.-Androstanedione	222865	C[C@]12CCC(=O)C[C@@H]1CC[C@@H]3[C@@H]2CC[C@]4([C@H]3CCC4=O)C
Hesperetin	72281	COC1 = C(C = C(C = C1)[C@@H]2CC(=O)C3 = C(C = C(C = C3O2)O)O)O
Amentoflavone	5281600	C1 = CC(=CC = C1C2=CC(=O)C3 = C(O2)C(=C(C = C3O)O)C4 = C(C = CC(=C4)C5 = CC(=O)C6 = C(C = C(C = C6O5)O)O)O)O
Luteolin	5280445	C1 = CC(=C(C = C1C2=CC(=O)C3 = C(C = C(C = C3O2)O)O)O)O
Tyrosol	10393	C1 = CC(=CC = C1CCO)O
Syringic acid	10742	COC1 = CC(=CC(=C1O)OC)C(=O)O
Luteolin 7-O-neohesperidoside	5282152	C[C@H]1[C@@H]([C@H]([C@H]([C@@H](O1)O[C@@H]2[C@H]([C@@H]([C@H](O[C@H]2OC3=CC(=C4C(=C3)OC(=CC4 = O)C5 = CC(=C(C = C5)O)O)O)CO)O)O)O)O)O
n-Hexadecanoic acid	985	CCCCCCCCCCCCCCCC(=O)O
Heneicosanoic acid	16898	CCCCCCCCCCCCCCCCCCCCC(=O)O
9, 12-Octadecadienoic acid	5280450	CCCCC/C = C\C/C = C\CCCCCCCC(=O)O
Eicosanoic acid	10467	CCCCCCCCCCCCCCCCCCCC(=O)O
Squalene	638072	CC(=CCC/C(=C/CC/C(=C/CC/C = C(/CC/C = C(/CCC = C(C)C)\C)\C)/C)/C)C
Heptadecanoic acid	10465	CCCCCCCCCCCCCCCCC(=O)O
Tetramethyl-2- hexadecen-1-ol	5280435	C[C@@H](CCC[C@@H](C)CCC/C(=C/CO)/C)CCCC(C)C
Terpenoids	221493	C[C@H](CCC(=O)O)[C@H]1CC[C@@H]2[C@@]1([C@H](C[C@H]3[C@H]2[C@@H](C[C@H]4[C@@]3(CC[C@H](C4)O)C)O)O)C
Benzoic acid, 2-chloro-	8374	C1 = CC = C(C(=C1)C(=O)O)Cl
4-Oxo-2-thioxo-3-thiazolidineacetic acid, ethyl ester	141009	CCOC(=O)CN1C(=O)CSC1 = S
Fluometuron	16562	CN(C)C(=O)NC1 = CC = CC(=C1)C(F)(F)F
3-Tropoyloxy-6-acetoxytropane	91750092	CC(=O)OC1C[C@H]2CC(C[C@@H]1N2C)OC(=O)C(CO)C3 = CC = CC = C3
Otosenine	6438142	C[C@@H]1C[C@]2([C@@H](O2)C)C(=O)O[C@@H]3CCN(C/C = C(\C3 = O)/COC(=O)[C@]1(C)O)
Dichlorphen, O,O’-di(pentafluorobenzoyl)-	91732176	C1 = CC(=C(C = C1Cl)CC2 = C(C = CC(=C2)Cl)OC(=O)C3 = C(C(=C(C(=C3F)F)F)F)F)OC(=O)C4 = C(C(=C(C(=C4F)F)F)F)F
Heptanoic acid, 6-oxo-	98810	CC(=O)CCCCC(=O)O
Benzenesulfonamide, N,4-dimethyl-N-phenyl-	220023	CC1 = CC = C(C = C1)S(=O)(=O)N(C)C2 = CC = CC = C2
Naftidrofuryl	4417	CCN(CC)CCOC(=O)C(CC1CCCO1)CC2 = CC = CC3 = CC = CC = C32
Trifluperidol	5567	C1CN(CCC1(C2 = CC(=CC = C2)C(F)(F)F)O)CCCC(=O)C3 = CC = C(C = C3)F
5-Quinolinesulfonic acid, 8-hydroxy-7-(6-sulfo-2-naphthylazo)-	92577	C1 = CC2 = C(C = C(C(=C2N=C1)O)N = NC3 = CC4 = C(C = C3)C = C(C = C4)S(=O)(=O)O)S(=O)(=O)O
Hexanedioic acid, 2-[(tert-butyldimethylsilyl)amino]-	553979	CC(C)(C)[Si](C)(C)NC(CCCC(=O)O[Si](C)(C)C(C)(C)C)C(=O)O[Si](C)(C)C(C)(C)C
Glutaric acid, 2-chloro-6-fluorophenyl pentachlorophenyl ester	91692769	C1 = CC(=C(C(=C1)Cl)OC(=O)CCCC(=O)OC2 = C(C(=C(C(=C2Cl)Cl)Cl)Cl)Cl)F
Dopamine, N-DTFMB-TMS	526013	C[Si](C)(C)OC1 = C(C = C(C = C1)CCNC(=O)C2 = CC(=CC(=C2)C(F)(F)F)C(F)(F)F)O[Si](C)(C)C
Hydrocinnamic acid, 4-(4-hydroxy-3-iodophenoxy)-3,5-diiodo-	5804	C1 = CC(=C(C = C1OC2=C(C = C(C = C2I)CCC(=O)O)I)I)O
Anthralin, O,O’,O’‘-tri(pentafluoropropionyl)-	91745919	C1 = CC2 = CC3 = C(C(=CC = C3)OC(=O)C(C(F)(F)F)(F)F)C(=C2C(=C1)OC(=O)C(C(F)(F)F)(F)F)OC(=O)C(C(F)(F)F)(F)F
2-Hexenoic acid, methyl ester	61310	CCCC = CC(=O)OC
Propanamide, N,N-dihexyl-2-methyl-	532799	CCCCCCN(CCCCCC)C(=O)C(C)C
Retinol	445354	CC1=C(C(CCC1)(C)C)/C = C/C(=C/C = C/C(=C/CO)/C)/C
Isonipecotic acid	3773	C1CNCCC1C(=O)O
Norepinephrine, N-isoBOC, O-TBDMS	91749314	CC(C)COC(=O)NCC(C1 = CC(=C(C = C1)O[Si](C)(C)C(C)(C)C)O[Si](C)(C)C(C)(C)C)O[Si](C)(C)C(C)(C)C
p-hydroxybenzyl glucosinolate, TMS	91748941	C[Si](C)(C)OCC1C(C(C(C(O1)SC(=NOS(=O)(=O)O[Si](C)(C)C)CC2 = CC = C(C = C2)O[Si](C)(C)C)O[Si](C)(C)C)O[Si](C)(C)C)O[Si](C)(C)C
Propanamide, N,N-didecyl-3-phenyl-	532928	CCCCCCCCCCN(CCCCCCCCCC)C(=O)CCC1 = CC = CC = C1
l-Phenylalanine, n-pentafluoropropionyl-, hexadecyl ester	91719463	CCCCCCCCCCCCCCCCOC(=O)C(CC1 = CC = CC = C1)NC(=O)C(C(F)(F)F)(F)F
Xylitol, pentakis(trifluoroacetate	550569	C(C(C(C(COC(=O)C(F)(F)F)OC(=O)C(F)(F)F)OC(=O)C(F)(F)F)OC(=O)C(F)(F)F)OC(=O)C(F)(F)F
Linalool, Gly, TFA	91749418	CC(=CCCC(C)(C = C)O[C@@H]1[C@H]([C@@H]([C@H]([C@@H](O1)COC(=O)C(F)(F)F)OC(=O)C(F)(F)F)OC(=O)C(F)(F)F)OC(=O)C(F)(F)F)C

*In Silico Virtual Screening Against Sucrase, Alpha Amylase, and Glucosidase Enzymes section:*

### Molecular dynamics simulation

Molecular dynamics (MD) simulations were performed on the selected amylase luteolin, sucrase luteolin, and α-glucosidase luteolin complexes for 60 ns using OpenMM. Each system was solvated in an explicit TIP3P water box with a minimum distance of 10 Å between the protein surface and the box boundary. Protein topologies were generated using AmberTools with the ff19SB force field, while ligand parameters for luteolin were generated using the General Amber Force Field (GAFF2) with AM1-BCC partial charges [19].

RMSD analysis was performed on protein backbone atoms (Cα) after least-squares fitting to the initial backbone conformation. Radius of gyration (RoG) was calculated for the protein to evaluate global compactness during the simulation. Furthermore, per-residue RMSF analyses were included to evaluate local flexibility and to identify residues contributing to binding stability at the active site. These analyses indicate limited fluctuations of catalytic and binding site residues throughout the simulation, supporting stable ligand binding.

All systems were neutralized and ionized with 0.15 M NaCl to mimic physiological ionic strength. Energy minimization was carried out for 20,000 steps using the steepest descent algorithm. Subsequently, the systems were equilibrated for 2 ns under NPT conditions at 298 K and 1 atm (101,325 Pa), applying a positional restraint of 500 kJ/mol/nm² on protein backbone atoms. Temperature was maintained using a Langevin thermostat with a collision frequency of 1 ps ⁻ ¹, and pressure was controlled using a Monte Carlo barostat. Long-range electrostatic interactions were treated using the Particle Mesh Ewald (PME) method with a real-space cutoff of 10 Å, while van der Waals interactions employed a cutoff of 10 Å. A 2 fs integration time step was used, and all covalent bonds involving hydrogen atoms were constrained using the SHAKE algorithm.

Visualization and graphical representation were accomplished using Matplotlib, Paint.NET, and the open-source PyMOL software. All simulations and analyses were performed on an Nvidia V100 GPU within Google Colab [20].

MM-PBSA and MM-GBSA binding free energy calculations were performed using a single trajectory approach, which is widely employed for comparative binding affinity estimation in protein ligand systems. This approach was minimizes structural noise by using identical conformational ensembles for complex, receptor, and ligand, thereby improving internal consistency. The binding free energy values were therefore interpreted in a relative (ranking-based) manner rather than as absolute thermodynamic quantities.

### Docking scoring and statistical analysis

For each ligand receptor system, AutoDock Vina generated multiple binding poses, which were evaluated based on predicted binding free energies and pose geometry. The lowest energy pose with a root mean square deviation (RMSD) ≤ 2.0 Å relative to the top-ranked conformation was selected for subsequent analyses. Docking scores are reported as predicted binding free energies (kcal/mol) calculated using the AutoDock Vina scoring function, where more negative values indicate stronger predicted binding affinity. To account for pose variability and ensure robustness of ligand ranking, binding energies across the highest-scoring conformations were compared for each ligand–receptor complex. Further, molecular docking provides relative rather than absolute estimates of binding affinity, results were interpreted comparatively across ligands and targets rather than subjected to inferential statistical testing.

## Results and discussion

### Identification of bioactive compounds by LC ESI MS/MS section

**Fig 1:**
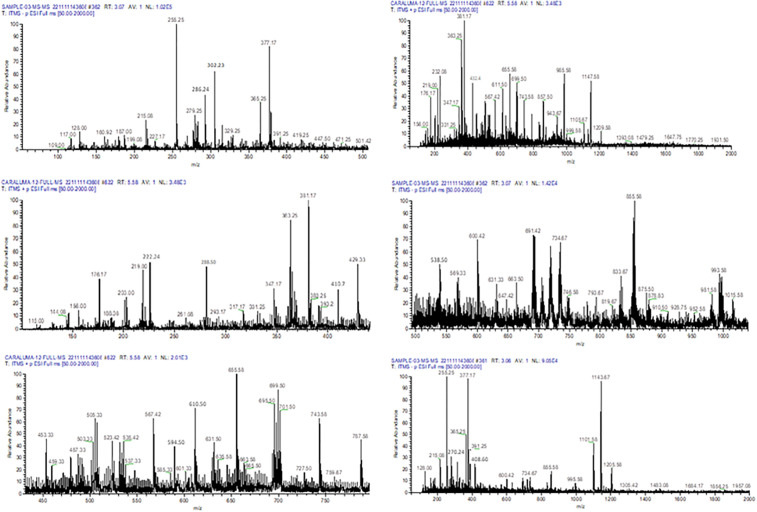
Mass spectrum of bioactive compounds in Caralluma tuberculata methanolic extracts analyzed by liquid chromatography electrospray ionization-tandem mass spectrometry (LC-ESI-MS/MS). Please use panels for each figure and describe the identified molecule.

Liquid chromatography coupled with mass spectrometry (LC-MS) is a highly effective analytical tool for organic compound identification [21]. Traditionally, Caralluma species have been widely used in herbal medicine for managing diabetes mellitus (DM). To identify potential bioactive antidiabetic metabolites in *Caralluma tuberculata*, we employed the LC/ESI-MS/MS technique. Our analysis revealed 57 potent antidiabetic compounds within the fractions, with 50% methanol used as the solvent (Fig 1), including metabolites such as rutin, kaempferol, and luteolin (Table 1). The presence of these bioactive antidiabetic compounds may be attributed to the plant’s defensive mechanisms for scavenging oxidative species under stress conditions [22]. The identified polyphenols and antidiabetic compounds likely play an essential role in counteracting reactive oxygen species, contributing to their beneficial effects [9].

Previously, no comprehensive LC ESI MS/MS profiling of *Caralluma tuberculata* for antidiabetic properties was available. However, LC-MS/MS analysis of polyphenolic extracts from C. europaea has identified similar phenolic compounds, such as kaempferol, luteolin, and kaempferol-3-O-hexose-deoxyhexose [23]. In other research, the phytoconstituents of *Caralluma tuberculata* were quantified using peak areas of each m/z value, as indicated by [24]. Their LC-MS analysis of methanolic extract (ME) identified compounds like russelioside B, russelioside C, and caratuberside C. Previous reports suggest that such metabolites enhance lipid uptake, improve glucose absorption, and regulate blood sugar—key factors in blood glucose management [25]. This study’s findings on *Caralluma tuberculata* metabolites hold potential as significant antidiabetic agents and offer valuable bi]oactive resources for pharmaceutical applications.

Molecular docking is a valuable tool for predicting interactions between small molecules and target proteins, serving as a key step in drug discovery and design. To efficiently screen the numerous natural compounds extracted from *Caralluma tuberculata*, molecular docking provides a rapid approach. Docking allows rapid, structure based evaluation of binding modes and relative affinities of multiple ligands toward the catalytic sites of α-amylase, sucrase, and α-glucosidase. This strategy enables efficient prioritization of lead compounds for subsequent molecular dynamics simulations and free energy calculations, which were then applied to the top-ranked candidate. This approach is widely used in natural-product based drug discovery to efficiently narrow down lead compounds prior to experimental validation [26]. Identifying natural inhibitors of carbohydrate-hydrolyzing enzymes alpha-amylase, sucrase, and alpha-glucosidase is essential, as these enzymes play pivotal roles in breaking down complex sugars [27]. Inhibiting these enzymes is a significant strategy for managing postprandial hyperglycemia, a common complication in diabetes mellitus (DM) [28] Enzyme inhibition slows carbohydrate digestion, decreases glucose absorption, and ultimately moderates post-meal blood glucose spikes [29]. Conventional antidiabetic drugs like acarbose, voglibose, and miglitol are effective but often produce undesirable side effects, including bloating and gastrointestinal issues [30]. These drugs are frequently associated with gastrointestinal adverse effects, largely due to their mechanism of delaying carbohydrate digestion in the intestine [31]. Acarbose voglibose, and miglitol treatment resulted in a significantly higher incidence of gastrointestinal events including flatulence, abdominal bloating, and diarrhea and these side effects were dose-dependent and often led to reduced treatment compliance [32]. This has led to increased interest in natural alternatives, with ethnopharmacological sources documenting over 1,200 plants with antidiabetic properties [33].

Natural compounds such as plant flavonoids and other phytochemicals may differ in their side effect profile due to intrinsic pharmacokinetic and pharmacodynamic properties. plant-derived metabolites exhibit moderate mechanisms of action through multiple pathways, including decreased hepatic glucose production, inhibition of carbohydrate-digesting enzymes modulation of metabolic regulators such as PPARγ, hypolipidemic and antioxidant activities, inhibition of glycolytic enzymes including phosphoenolpyruvate carboxykinase, reduction of glycosylated hemoglobin levels, and enhanced expression of glucose transporters rather than potent inhibition of a single enzyme, compared to synthetic inhibitors [34]. Additionally, natural compounds often have historical dietary exposure and traditional use, suggesting generally better tolerability and safety in human populations, although rigorous comparative clinical data are limited [35]. These features make phytochemicals attractive candidates for further study as antidiabetic agents with potentially lower incidence of undesirable effects, supporting the rationale for their investigation.

This study aims to discover potential natural inhibitors from *Caralluma tuberculata* using molecular docking and dynamic simulation methods. The consistency of predicted binding orientations and interaction profiles with those of native co crystallized ligands confirms the reliability and validity of the applied docking protocol. This approach is both efficient and accurate, enabling rapid screening of extensive compound libraries and providing detailed interaction profiles at the atomic level. A library of 57 bioactive compounds isolated from *Caralluma tuberculata* was screened against alpha-amylase, sucrase, and alpha-glucosidase through molecular docking. Luteolin (PubChem CID: 5280445) emerged as the top candidate, showing strong binding affinities with energy scores of −9.725, −8.19, and −7.842 kcal/mol for alpha-amylase, sucrase, and alpha-glucosidase, respectively (Table 2,3,4). These scores indicate notable inhibitory potential, aligning with findings in existing studies. For example, Luteolin’s binding affinity of −8.6 kcal/mol has been previously recorded in enzyme inhibition studies using similar methods [36]. Additionally, research by [37] supports the efficacy of phytochemicals like Luteolin in inhibiting alpha-amylase and alpha-glucosidase, further validating our results.

**Table 2:** Molecular Docking of Compounds with Alpha-amylase top (n = 10) compounds were chosen based on the best docking scores.

Compounds Name	Pubchem CID	Dock Score Binding affinity predicted by AutoDock Vina (kcal/mol)	LABO RMSD Root-mean-square deviation (Å) of docked pose relative to the reference ligand calculated using the LaBOX method	Target	Toxicity Risk	cLogP (Consensus logP (lipophilicity)	logS (Aqueous solubility prediction)	MV (Molecular volume (Å³)	TPSA (Topological polar surface area (Å²)	DL (Drug-likeness score)	DS (Drug score integrating physicochemical and toxicity parameters)
Quercetin	5280343	−9.764	6.919	Amylase	Predicted Mutaginc, Tumerigenic	1.49	−2.5	302	127.4	1.6	0.3
Luteolin	5280445	−9.725	6.897	Amylase	No Risk	1.99	−2.6	286	107.2	1.9	0.84
5. alpha.-Androstanedione	222865	−9.215	4.959	Amylase	Predicted Reproductive Effect	3.74	−4.4	288	34.14	−0.33	0.3
Pregnane	439513	−9.12	4.984	Amylase	Predicted Reproductive Effect	5.88	−5.5	288	0	−4.88	0.17
Amentoflavone	5281600	−9.083	8.229	Amylase	No Risk	4.67	−6.2	538	173.9	2.03	0.32
Cholic acid	221493	−8.916	7.008	Amylase	No Risk	3.18	−4.5	408	97.99	0.24	0.52
Emodin	3220	−8.815	3.904	Amylase	Predicted Mutagenic, Tumerigenic, Irritant, Reproductive Effect	2.34	−4.2	270	94.83	−2.06	0.06
Kaempferol	5280863	−8.728	6.917	Amylase	Predicted Mutegenic	0.77	−3.2	432	166.1	2.76	0.46
Dichlorphen	3037	−8.67	3.711	Amylase	No Risk	8.56	−11	656	52.6	−9.73	0.07
Flavone	10680	−8.599	6.476	Amylase	Predicted Mutagenic	3.37	−3.7	222	26.3	1.85	0.45

**Table 3:** Molecular Docking of Compounds with Sucrase top (n = 10) compounds were chosen based on the best docking scores.

Compounds Name	Pubchem CID		Dock Score Binding affinity predicted by AutoDock Vina (kcal/mol)	LABO RMSD Root-mean-square deviation (Å) of docked pose relative to the reference ligand calculated using the LaBOX method	Target	Toxicity Risk	cLogP (Consensus logP (lipophilicity)	logS (Aqueous solubility prediction)	MV (Molecular volume (Å³)	TPSA (Topological polar surface area (Å²)	DL (Drug-likeness score)	DS (Drug score integrating physicochemical and toxicity parameters)
Flavone	10680	−8.517		3.85	Sucrase	Mutaginc	3.37	−3.74	222	26.3	1.85	0.45
Dopamine	526013	−8.431		3.046	Sucrase	Irritant,	8.34	−7.09	537	47.56	−69.33	0.06
Rutin	5280805	−8.367		4.443	Sucrase	No Risk	−1.26	−2.4	610	265.5	3.31	0.57
Luteolin	5280445	−8.19		7.334	Sucrase	No Risk	1.99	−2.56	286	107.2	1.9	0.84
Trifluperidol	5567	−8.098		6.172	Sucrase	No Risk	1.99	−2.56	286	107.2	1.9	0.84
Quercetin	5280343	−8.051		6.956	Sucrase	Mutagenic, Tumerigenic	1.49	−2.49	302	127.4	1.6	0.299
Quinolinesulfonic acid	22506530	−8.021		6.649	Sucrase	Mutagenic, Tumerigenic, Irritant, Reproductive Effect	0.57	−3.34	459	183.3	−7.65	0.06
Kaempferol	5280863	−7.864		7.883	Sucrase	Mutegenic	0.77	−3.18	432	166.1	2.76	0.46
Naftidrofuryl	4417	−7.639		4.812	Sucrase	Mutegenic, Irritant	4.3	−4.44	383	38.77	6.21	0.22
Amentoflavone	5281600	−7.618		5.63	Sucrase	No Risk	−6.	−6.18	538	173.9	2.03	0.32

**Table 4:** Molecular Docking of Compounds with Alpha-glucosidase top (n = 10) compounds were chosen based on the best docking scores.

Compounds Name	Pubchem CID	Dock Score Binding affinity predicted by AutoDock Vina (kcal/mol)	LABO RMSD Root-mean-square deviation (Å) of docked pose relative to the reference ligand calculated using the LaBOX method	Target	Toxicity Risk	cLogP (Consensus logP (lipophilicity)	logS (Aqueous solubility prediction)	MV (Molecular volume (Å³)	TPSA (Topological polar surface area (Å²)	DL (Drug-likeness score)	DS (Drug score integrating physicochemical and toxicity parameters)
Amentoflavone	5281600	−8.612	9.888	Glucosidase	No Risk	4.67	−6.18	538	173.9	2.03	0.32
Kaempferol	5280863	−8.534	2.773	Glucosidase	Mutagenic	0.77	−3.18	432	166.1	2.76	0.46
Dopamine	526013	−8.291	8.176	Glucosidase	Irritant	8.34	−7.09	537	47.56	−69.33	0.06
Flavone	10680	−7.926	7.237	Glucosidase	Mutagenic	3.37	−3.74	222	26.3	1.85	0.45
Luteolin	5280445	−7.842	3.317	Glucosidase	No Risk	1.99	−2.56	286	107.2	1.9	0.84
Trifluperidol	5567	−7.826	5.042	Glucosidase	Reproductive Effect	4.55	−4.74	409	40.54	5.28	0.33
Kaempferol	5280863	−7.805	2.774	Glucosidase	Mutagenic	−1.06	−2.88	594	245.2	−1.35	0.21
Quercetin	5280343	−7.778	8.301	Glucosidase	Mutagenic, Tumerigenic	1.49	−2.49	302	127.4	1.6	0.299
Squalene	638072	−7.707	10.791	Glucosidase	No Risk	13.1	−6.3	410	0	−3.52	0.14
Dichlorphen	3037	−7.702	9.605	Glucosidase	No Risk	8.56	−10.88	656	52.6	−9.73	0.07

**Fig 2:**
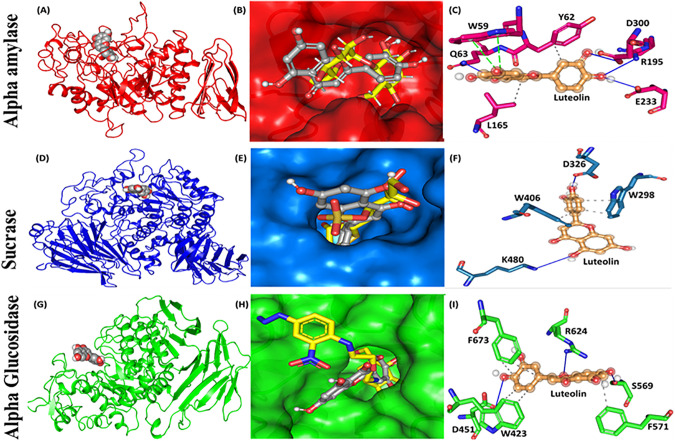
Luteolin, attachment in the active-pocket regions of all tree receptors. Alpha amylase (A,B,C); Sucraase (D,E,F); Alpha Glucosidase (G,H,I).

A detailed examination of enzyme-ligand interactions revealed that Luteolin engages with essential residues that are crucial for the catalytic activities of these enzymes. In alpha-amylase, Luteolin forms hydrogen bonds with residues D195, D300, and E233, which are recognized for their roles as nucleophiles and acid/base catalysts in substrate hydrolysis [38]. These interactions are essential for effective enzyme inhibition, as shown by the crystal structure of alpha-amylase bound to Luteolin, which highlights substantial binding within the enzyme’s active site. Comparable interactions were identified in sucrase (D326 and K480) and alpha-glucosidase (D451 and R624), where Luteolin assumed a similar orientation to the control ligand (Fig 2). Additionally, π-stacking interactions and van der Waals forces contributed to the stability of the complex, especially in alpha-amylase and sucrase, enhancing Luteolin’s binding affinity. These critical interactions suggest that Luteolin may be effective in inhibiting alpha-amylase and sucrase, thus slowing carbohydrate breakdown and glucose absorption. This finding aligns with previous studies on Luteolin’s pharmacological properties, which include antidiabetic, anti-inflammatory, and antioxidant effects [39].

Before progressing to in vivo testing, assessing the pharmacokinetic properties of any candidate compound is crucial to confirm its drug suitability. In silico analysis of Luteolin’s pharmacokinetics revealed compliance with Lipinski’s rule of five, a fundamental measure of drug-likeness. Its total polar surface area (TPSA) of 107.2 Å² indicates good membrane permeability, complemented by a drug score of 0.84 and a drug-likeness value of 1.9, which collectively predict a favorable safety profile with no significant toxicity concerns. These findings align with study [40], which highlight the promising pharmacokinetic attributes of flavonoids as antidiabetic agents. Toxicity evaluations, an essential aspect of drug discovery, showed that Luteolin posed no risk of adverse effects across all enzyme targets. Computational toxicity assessments predicted its safety profile, with no signs of hepatotoxicity, mutagenicity, or carcinogenicity. Paired with its strong inhibitory profile, these results position Luteolin could be potential candidate for further drug development. However, as the docking process relies on static protein structures, it cannot fully replicate the binding behavior in dynamic biological environments. Consequently, we conducted simulations for each protein Luteolin complex to further validate these findings.

**Fig 3:**
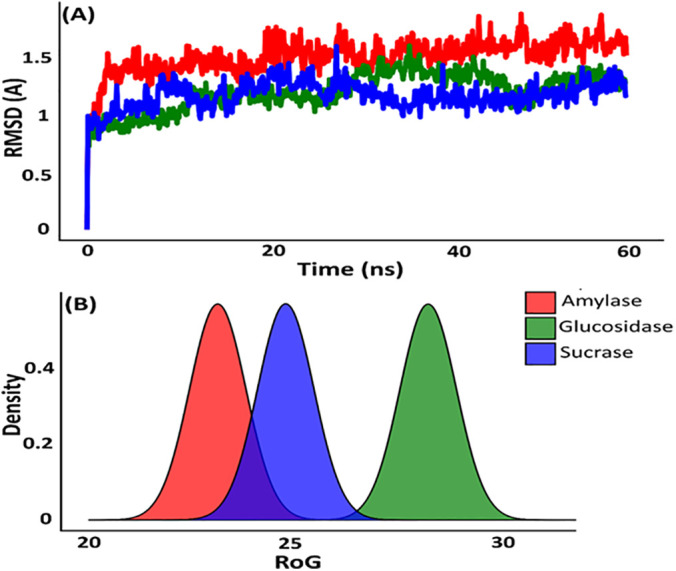
Molecular dynamics (MD) simulation of the protein-ligand complex.

To assess the stability of the protein-ligand complexes, molecular dynamics (MD) simulations were performed over 60 ns for each enzyme-Luteolin complex. The results showed that Luteolin maintained stable interactions throughout, with minimal fluctuations in root-mean-square deviation (RMSD) values. Alpha-amylase and sucrase complexes displayed particularly stable binding, confirming that Luteolin remains securely bound within the active sites over time (Fig 3). Although alpha-glucosidase showed slightly less stability, its interactions were still promising and may benefit from further structural optimization. Luteolin’s inhibitory potential against alpha-amylase, sucrase, and alpha-glucosida se underscores its suitability for antidiabetic drug development. Its ability to inhibit these enzymes through stable binding and favorable pharmacokinetic properties highlights its therapeutic potential [41]. While molecular docking and dynamics simulations provide valuable insights, they are limited by reliance on static protein models. Nonetheless, integrating molecular docking, dynamic simulations, and pharmacokinetic profiling forms a comprehensive approach that can accelerate drug discovery, paving the way for novel natural antidiabetic therapies [42].

Molecular dynamics (MD) simulations offer a powerful approach to examining the dynamic behavior and stability of protein-ligand complexes over time. By simulating interactions between proteins and ligands in a virtual setting, MD simulations provide valuable insights into conformational shifts, binding stability, and molecular flexibility occurring during ligand-target protein interactions [43]. These simulations not only reveal structural gaps that may be optimized for designing more potent inhibitors but also enable a more precise estimation of binding affinities, thereby enhancing drug design accuracy [44]. In this study, we conducted 60 ns MD simulations on the complexes of Luteolin with alpha-amylase, sucrase, and alpha-glucosidase, following molecular docking experiments. This analysis aimed to assess the structural dynamics and stability of each complex, with a focus on root mean square deviation (RMSD), radius of gyration (RoG), and thermodynamic parameters, including total energy, van der Waals (vdW) energy, and electrostatic energy. These metrics are essential for understanding protein-ligand interactions within a dynamic biological environment [45]. Throughout the 60 ns molecular dynamics simulations, the protein backbone RMSD, calculated after least squares fitting to the backbone atoms, showed rapid equilibration within the initial phase of the simulations and remained stable thereafter for all three protein luteolin complexes. The RMSD values fluctuated within a narrow range indicating overall structural stability of the protein frameworks during the simulation period. The absence of large RMSD fluctuations suggests that no major conformational rearrangements occurred in the protein structures upon ligand binding. While RMSD primarily reflects global protein stability rather than binding strength, the observed stable backbone trajectories, together with consistent radius of gyration profiles, support the maintenance of stable protein ligand complexes throughout the simulations.

**Fig 4:**
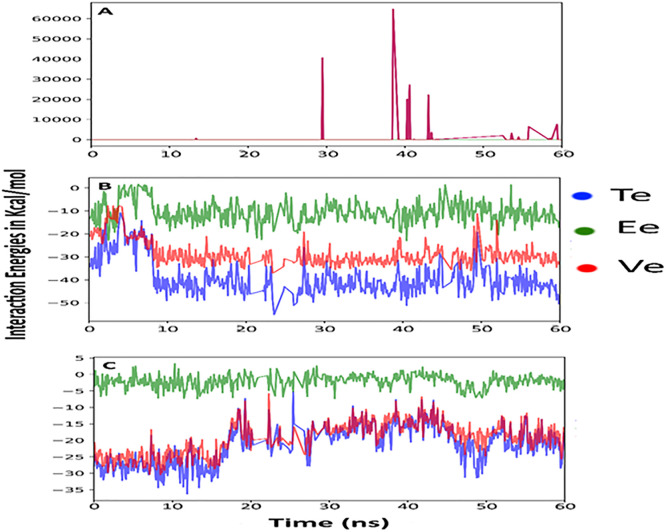
Overall system energies (Total energy, Van Der Waal energy, and Electrostatic energy).

RoG analysis sheds light on the compactness or expansion of each protein-ligand complex over time [46]. In our study, RoG profiles showed slight differences in the compactness of the three complexes: while the Luteolin complexes with alpha-amylase and sucrase maintained relatively compact structures, the glucosidase complex exhibited gradual expansion, as indicated by an increasing RoG value. This expansion suggests increased flexibility in the glucosidase binding site, which could provide further opportunities to enhance ligand binding affinity through structural optimization of Luteolin (Fig 3). Energy contributions to the stability of the complexes were examined by analyzing total energy, vdW energy, and electrostatic energy. Results indicated that the stability of Luteolin-protein complexes was predominantly driven by electrostatic interactions, which acted as the main stabilizing force across all three complexes. This finding aligns with the charged nature of amino acids involved in Luteolin binding, especially those within the catalytic sites of alpha-amylase, sucrase, and glucosidase. Notably, the alpha-amylase-Luteolin complex showed occasional vdW energy spikes around the 30 ns and 40 ns time points, likely due to temporary repulsive interactions with hydrophobic residues in the amylase binding pocket. Despite these minor fluctuations, strong electrostatic interactions offset vdW repulsions, ensuring that Luteolin remained stably bound (Fig 4). The molecular flexibility observed in the glucosidase-Luteolin complex, as evidenced by the RoG data, could be significant for designing more potent inhibitors. The expansion of the binding site might allow for additional binding interactions, which could be exploited by modifying Luteolin’s structure or creating analogs with enhanced binding affinity. This flexibility in glucosidase could also support multiple binding poses, potentially benefiting drug efficacy [47].

The MD simulations performed in this study offer key insights into the dynamic stability and behavior of Luteolin as a prospective inhibitor of alpha-amylase, sucrase, and alpha-glucosidase. The stable RMSD profiles across all three complexes suggest that Luteolin forms robust interactions with these enzymes. Meanwhile, RoG and energy analyses reveal subtle distinctions in the structural dynamics of each complex, particularly highlighting the more flexible nature of the glucosidase binding site, which presents opportunities for further optimization of Luteolin to enhance its inhibitory activity.

The molecular mechanics/generalized Poisson–Boltzmann or Born surface area (MM-PBSA and MM-GBSA) methods are essential tools for predicting protein-ligand binding free energies, especially in structure-based drug design. These approaches balance computational efficiency with precision, making them effective for estimating end-point binding free energies [48]. MM/GBSA, in particular, is often favored for its higher accuracy over traditional molecular docking scoring, positioning it as a valuable method in drug discovery [38].

Using these methods, we applied MM-PBSA and MM-GBSA techniques to calculate the binding free energies of Luteolin with alpha-amylase, sucrase, and alpha-glucosidase, thus gaining insights into the stability and interaction dynamics of these complexes over a 60 ns molecular dynamics simulation. Importantly, the relative binding trends obtained from MM-PBSA and MM-GBSA analyses were consistent across all three enzyme–luteolin complexes, and these trends correlated well with docking scores and MD stability metrics (RMSD, RMSF, and Rg), supporting the robustness of the comparative analysis. Our binding free energy calculations revealed notable variations in Luteolin’s interaction strengths with the three enzymes. Alpha-amylase and sucrase displayed more favorable binding free energy values than alpha-glucosidase. Specifically, MM-GBSA results showed binding free energies of −25.6712 and −23.9563 kcal/mol for alpha-amylase and sucrase, respectively, while MM-PBSA values were −0.9687 and −2.3314 kcal/mol. These favorable energy values indicate strong, stable interactions with these enzymes, underscoring Luteolin’s potential as an inhibitor of carbohydrate-digesting enzymes (Tables 5, 6). In contrast, the binding free energy for the Luteolin-alpha-glucosidase complex was less favorable, with MM-GBSA and MM-PBSA values of −13.2557 and 7.0167 kcal/mol, respectively. Analyzing the simulation trajectories revealed a destabilizing effect in the Luteolin-glucosidase complex, likely due to a slight separation between the N- and C-terminal domains of glucosidase, which enclose the active site. This movement, along with the flipping of a loop region (residues S623 to F627), disrupted a critical hydrogen bond with R624, causing Luteolin to exit the binding pocket after approximately 20 ns **(Video uploaded about Luteolin and alphaglucosidase interaction supporting information**

**Table 5:** Molecular mechanics/generalized Poisson–Boltzmann surface area MM/GBSA) approach for calculating protein-ligand binding free energies.

Compound	Analysis	EvdW	Eel	EGB	ESURF	Ggas	Gsolv	Total
**Luteolin**	Complex (G_SL_)	−7675.2707	−59381.3273	−11124.8422	211.6629	−67056.5979	−10913.1793	−77969.7772
	Complex (G_AL_)	−4292.3712	−35799.5720	−4522.1137	117.8569	−40091.9433	−4404.2568	−44496.2001
	Complex (G_GL_)	−5032.8652	−38648.0708	−7001.3411	161.8069	−43680.9360	−6839.5342	−50520.4702
	Receptor (G_S_)	−7641.7459	−59400.1049	−11140.5250	212.4872	−67041.8509	−10928.0378	−77969.8886
	Receptor (G_A_)	−4260.7814	−35818.3536	−4533.3797	119.1123	−40079.1350	−4414.2674	−44493.4024
	Receptor (G_G_)	−5010.2054	−38663.3840	−6999.2409	161.6333	−43673.5895	−6837.6076	−50511.1971
	Ligand (G_SL_)	−3.1902	39.4841	−14.8949	2.6687	36.2939	−12.2262	24.0677
	Ligand (G_AL_)	−3.1474	36.9922	−13.6394	2.6681	33.8448	−10.9712	22.8735
	Ligand (G_GL_)	−3.0670	19.0945	−14.7160	2.6710	16.0274	−12.0450	3.9825
	**Difference (ΔG**_**SL-bind**_)	−30.3345	−20.7064	30.5777	−3.4930	−51.0410	27.0847	**−23.9563**
	**Difference (ΔG**_**AL-bind**_)	−28.4424	−18.2106	24.9054	−3.9236	−46.6530	20.9818	**−25.6712**
	**Difference (ΔG**_**GL-bind**_)	−19.5928	−3.7812	12.6158	−2.4975	−23.3740	10.1183	**−13.2557**

**Table 6:** Molecular mechanics Born surface area (MM-PBSA) approach for calculating protein-ligand binding free energies.

Compound	Analysis	E_vdW_	E_el_	E_PB_	ENPOLAR	G_gas_	G_solv_	Total
**Luteolin**	Complex (G_SL_)	−7675.2707	−59381.3273	−10230.2164	6364.4122	−67056.5979	−6836.1606	−73892.7586
	Complex (G_AL_)	−4292.3712	−35799.5720	−4050.9141	3582.4011	−40091.9433	−2265.2685	−42357.2118
	Complex (G_GL_)	−5032.8652	−38648.0708	−6452.1198	4307.9528	−43680.9360	−4278.5643	−47959.5003
	Receptor (G_S_)	−7641.7459	−59400.1049	−10252.0448	6355.2698	−67041.8509	−6868.6021	−73910.4529
	Receptor (G_A_)	−4260.7814	−35818.3536	−4067.3156	3573.8919	−40079.1350	−2296.0485	−42375.1836
	Receptor (G_G_)	−5010.2054	−38663.3840	−6459.2521	4293.3147	−43673.5895	−4293.2758	−47966.8652
	Ligand (G_SL_)	−3.1902	39.4841	−13.6653	27.9027	36.2939	−16.2681	20.0258
	Ligand (G_AL_)	−3.1474	36.9922	−12.4578	27.9278	33.8448	−14.9043	18.9405
	Ligand (G_GL_)	−3.0670	19.0945	−13.0830	27.9679	16.0274	−15.6792	0.3482
	**Difference (ΔG**_**SL-bind**_)	−30.3345	−20.7064	35.4938	−18.7603	−51.0410	48.7096	**−2.3314**
	**Difference (ΔG**_**AL-bind**_)	−28.4424	−18.2106	28.8593	−19.4185	−46.6530	45.6843	**−0.9687**
	**Difference (ΔG**_**GL-bind**_)	−19.5928	−3.7812	20.2153	−13.3299	−23.3740	30.3907	**7.0167**

These results partially contrast with [49], who reported strong inhibitory effects of Luteolin on alpha-glucosidase. several factors may explain this discrepancy such as structure based computational models, differences in enzyme source and species, as α-glucosidase isoforms vary structurally between organisms, may significantly influence ligand binding behavior. Additionally, the crystallographic conformation of the α-glucosidase structure might be important to induced-fit effects occurring during experimental binding. Variations in protonation states of active-site residues, particularly under different pH conditions, can also alter hydrogen-bond networks and ligand orientation. Together, these factors likely contribute to the observed differences in binding affinity and stability, highlighting the need for complementary experimental validation. However, the structural instability of the binding pocket observed in our study may explain the lower binding affinity in the simulations. Optimization of Luteolin’s structure or modifications to the glucosidase binding site could enhance its stability and inhibitory efficacy against glucosidase. Further, longer simulations (>100 ns) could provide additional insight into slow, large-scale domain motions, particularly for α-glucosidase, and may further improve statistical robustness. Such long time scale simulations were beyond the scope of this screening oriented study and will be considered in future investigations.

**Fig 5:**
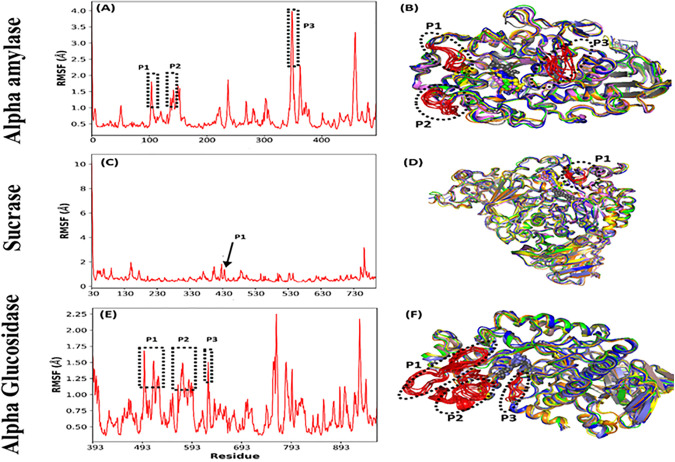
Root mean square fluctuation (RMSF) for all the receptors. Alpha amylase **(A,B)**; Sucrase **(C,D)**; Alpha Glucosidase **(E,F).**

To gain deeper insights into the conformational flexibility of the proteins during binding, we performed a root mean square fluctuation (RMSF) analysis on the protein-ligand complexes. RMSF measures the mobility of specific protein residues, which can significantly influence the stability of ligand-binding interactions [50]. The analysis revealed three distinct peaks in the RMSF profiles for alpha-amylase and glucosidase, and one peak for sucrase. In alpha-amylase, the peaks were located at regions G104-A109 (P1), D138-G143 (P2), and R346-D353 (P3), suggesting increased flexibility in these segments during the simulation. Similarly, glucosidase showed three peaks at V512-Y531 (P1), E567-H594 (P2), and S623-F627 (P3), with enhanced flexibility in the loop spanning S623 to F627 likely contributing to the destabilization of the Luteolin-glucosidase complex (Fig 5). For sucrase, a single peak was identified at E433-P436 (P1), indicating a more rigid structure overall compared to the other enzymes. The alignment of these fluctuating regions with the binding pocket highlights their importance in maintaining the stability of Luteolin binding. In alpha-amylase and sucrase, the flexibility observed did not appear to affect the binding stability of Luteolin; however, for glucosidase, the flexibility around the loop region S623-F627 directly impacted Luteolin’s ability to stay bound within the pocket.

These computational analyses provide valuable insights into the potential of Luteolin as a therapeutic agent for type 2 diabetes by targeting key carbohydrate-digesting enzymes. The strong binding interactions observed with alpha-amylase and sucrase suggest that Luteolin could serve as an effective inhibitor of these enzymes, potentially delaying carbohydrate digestion and reducing postprandial glucose spikes. Previous studies also have extensively documented the antidiabetic potential of luteolin through both experimental and computational approaches. In vivo investigations using streptozotocin induced diabetic rat models have demonstrated that luteolin significantly improves glycemic control, modulates lipid profiles, reduces oxidative stress and pro inflammatory cytokines, and ameliorates diabetes-associated tissue damage, with supportive evidence from molecular docking and molecular dynamics simulations [51]. Additionally, structure activity relationship studies have revealed that flavonoid (luteolin) are potent inhibitory activities supported by in vitro enzymatic assays, and molecular docking calculations [1]. Collectively, these findings establish luteolin and structurally related flavonoids as well characterized antidiabetic agents, providing a strong scientific foundation upon which the present computational analysis further builds. However, the decreased stability of the Luteolin-glucosidase complex points to the need for further optimization, specifically through structural adjustments to the glucosidase binding pocket, particularly around the S623-F627 loop, to enhance ligand-binding stability and improve inhibitory efficacy. In addition to binding energy calculations, we conducted ADMET (Absorption, Distribution, Metabolism, Excretion, and Toxicity) analyses of the phytochemicals isolated from *Caralluma tuberculata*. The broad range of physicochemical, pharmacokinetic, and drug-likeness properties of these compounds demonstrates that in-silico screening is a promising approach for identifying new drug candidates from natural sources [52]. Our MM-PBSA and MM-GBSA analyses confirmed the strong interactions between Luteolin and both alpha-amylase and sucrase, reinforcing their potential as therapeutic targets for type 2 diabetes. While Luteolin showed promising binding with these enzymes, its interactions with glucosidase were less stable, likely due to conformational shifts in the protein’s binding pocket. Future work should prioritize optimizing both the ligand and protein structures to enhance Luteolin’s inhibitory potential against glucosidase. These computational insights lay a foundation for the rational design of new antidiabetic compounds and underscore the critical role of structural flexibility in drug-receptor interactions.

## Conclusion

In this study, we investigated Luteolin, a compound identified from a selection of 57 compounds in *Caralluma tuberculata*, and demonstrated its in silico inhibition of alpha-amylase, sucrase, and alpha-glucosidase by binding to their active sites. LC/ESI-MS/MS analysis provided evidence of potent antidiabetic metabolites in *Caralluma tuberculata*. Molecular docking results revealed that Luteolin exhibited strong binding affinities and favorable drug-like properties, with binding energies of −9.725, −8.19, and −7.842 kcal/mol for alpha-amylase, sucrase, and alpha-glucosidase, respectively. In a 60 ns simulation, all proteins maintained a stable RMSD without irregular fluctuations, indicating a stable system. Analysis of total energy, van der Waals energy, and electrostatic energy showed that the complexes were primarily stabilized by electrostatic interactions. Binding energy calculations using MM-PBSA and MM-GBSA suggested that alpha-amylase and sucrase were the preferred receptors for Luteolin, with binding free energies of −25.6712 and −0.9687 kcal/mol for alpha-amylase, and −23.9563 and −2.3314 kcal/mol for sucrase, respectively, compared to alpha-glucosidase (−13.2557 and 7.0167 kcal/mol). Overall, the simulation analyses indicated that Luteolin formed stable complexes with alpha-amylase and sucrase, whereas its binding with alpha-glucosidase was less stable. These findings provide promising insights into developing Luteolin as a natural antidiabetic agent. Further animal trials are needed to validate and refine this phytochemical as an effective treatment option for type 2 diabetes management.

### Limitations of the study

Despite the promising computational findings, this study also have everal limitations. The inhibitory potential of luteolin against α-amylase, sucrase, and α-glucosidase was evaluated using *in silico* approaches, including molecular docking, molecular dynamics simulations, and MM-PBSA/MM-GBSA free energy calculations. These methods provide valuable insights into binding stability and interaction mechanisms, they do not substitute for experimental validation. The present MM-PBSA/MM-GBSA analysis was intended as a supportive and comparative tool to complement docking and molecular dynamics results rather than to provide absolute binding free energies. *In vivo* models are necessary to confirm the biological activity, bioavailability, and pharmacokinetic behavior of luteolin. Additionally, molecular dynamics simulations were limited to 60 ns and focused on single ligand–protein complexes, which may not fully capture long time scale conformational changes. Therefore, the conclusions of this study should be interpreted as predictive rather than definitive, serving as a foundation for future experimental and clinical investigations.

## Supporting information

S1 File Video visualization uploaded about Luteolin and alphaglucosidase interaction. (MP4)
